# The α2AR/Caveolin‐1/p38MAPK/NF‐κB axis explains dexmedetomidine protection against lung injury following intestinal ischaemia‐reperfusion

**DOI:** 10.1111/jcmm.16614

**Published:** 2021-06-10

**Authors:** Lin Xu, Taiyuan Li, Qiuhong Chen, Zhen Liu, Yuesheng Chen, Kai Hu, Xuekang Zhang

**Affiliations:** ^1^ Department of Anesthesiology The First Affiliated Hospital of Nanchang University Nanchang China; ^2^ Department of Gastrointestinal Surgery The First Affiliated Hospital of Nanchang University Nanchang China; ^3^ Department of Gastrointestinal Surgery The Fourth Affiliated Hospital of Nanchang University Nanchang China; ^4^ Nanchang Hongdu Hospital of traditional Chinese Medicine Nanchang China

**Keywords:** Caveolin‐1, dexmedetomidine, intestinal ischaemia‐reperfusion, lung injury, NF‐κB, p38MAPK, α_2A_‐AR

## Abstract

Intestinal ischaemia‐reperfusion (I/R) injury can result in acute lung injury due to ischaemia and hypoxia. Dexmedetomidine (Dex), a highly selective alpha2‐noradrenergic receptor (α2AR) agonist used in anaesthesia, is reported to regulate inflammation in organs. This study aimed to investigate the role and mechanism of Dex in lung injury caused by intestinal I/R. After establishing a rat model of intestinal I/R, we measured the wet‐to‐dry specific gravity of rat lungs upon treatments with Dex, SB239063 and the α2AR antagonist Atipamezole. Moreover, injury scoring and histopathological studies of lung tissues were performed, followed by ELISA detection on tumour necrosis factor‐α (TNF‐α), interleukin (IL)‐1β and IL‐6 expression. Correlation of Caveolin‐1 (Cav‐1) protein expression with p38, p‐p38, p‐p65 and p65 in rat lung tissues was analysed, and the degree of cell apoptosis in lung tissues after intestinal I/R injury was detected by TUNEL assay. The lung injury induced by intestinal I/R was a dynamic process. Moreover, Dex had protective effects against lung injury by mediating the expression of Cal‐1 and α_2A_‐AR. Specifically, Dex promoted Cav‐1 expression *via* α_2A_‐AR activation and mitigated intestinal I/R‐induced lung injury, even in the presence of Atipamezole. The protective effect of Dex on intestinal I/R‐induced lung injury was also closely related to α_2A_‐AR/p38 mitogen‐activated protein kinases/nuclear factor‐kappaB (MAPK/NF‐κB) pathway. Dex can alleviate pulmonary inflammation after in intestinal I/R by promoting Cav‐1 to inhibit the activation of p38 and NF‐κB. In conclusion, Dex can reduce pulmonary inflammatory response even after receiving threats from both intestinal I/R injury and Atipamezole.

## 1 INTRODUCTION

Intestinal ischaemia/reperfusion (I/R) injury is a common organ injury in surgical practice in the context of body ischaemia and hypoxia. Various pathological mechanisms can lead to the occurrence of intestinal I/R injury, including intestinal obstruction, severe trauma, intestinal transplantation, intestinal torsion, shock and thrombosis.^1^ The pathological mechanism of I/R involves various factors including oxidative stress caused by oxygen free radicals and the role of inflammatory cells, calcium overload and cell apoptosis.^2^, ^3^ Intestinal I/R injury can cause the release of intestinal toxins and bacteria, in addition intestinal oxygen free radicals and various inflammatory factors, causing downstream damage of other organs and systemic inflammatory reactions, the most prominent of which is lung damage.^4^ Acute lung injury (ALI) is a diffuse inflammatory pulmonary damage caused by excessive reactive oxygen species (ROS), which disrupt normal physiological structures and functions.^5^ The main pathogenesis of ALI is an inflammatory response mediated by various inflammatory cells cytokines released,^6^, ^7^ which can perturb mitochondrial metabolism and fission.^8^ When ALI occurs, the destruction of vascular endothelial cells and alveolar epithelial cells caused by inflammation may imperil normal respiratory function of the lungs, thus reducing the cellular oxygen partial pressure of the lung tissues, causing the body to experience hypoxia.^9^

In recent years, intestinal I/R‐related lung injury has received extensive attention as a clinically relevant research topic. Dexmedetomidine (Dex), a highly selective alpha2‐noradrenergic receptor (α2AR) agonist, is reported to have certain anti‐inflammatory and antioxidant effects, which can exert important protective effects in various organs and tissues.^10^, ^11^ Moreover, Dex can up‐regulate the expression of Caveolin‐1 (Cav‐1) in the lung tissues of the septic rat.^12^ Interestingly, Cav‐1 can suppress LPS‐induced phosphorylation of p38 mitogen‐activated protein kinases (MAPKs) and activation of the nuclear factor‐kappaB (NF‐κB) pathway, thus playing a protective role against lung injury.^13^, ^14^ However, the exact mechanism of the relationship between DEX and the p38MAPK/NF‐κB pathway in intestinal I/R‐induced lung injury has not been demonstrated.^13^ Based on the above research, we proposed that Dex may alleviate lung injury *via* regulating the α2AR/Cav‐1/p38MAPK/NF‐κB signalling axis. To test our hypothesis, we established a rat model of intestinal I/R injury to demonstrate the effect of DEX treatment on intestinal I/R‐induced lung injury mediated by the α2AR/Cav‐1/p38MAPK/NF‐κB axis.

## 2 MATERIALS AND METHODS

### 2.1 Animal model establishment of intestinal I/R injury

The Institutional Animal Care and Use Committee at The First Affiliated Hospital of Nanchang University approved all experimental procedures and protocols. Male Wistar rats (6 weeks, 208‐212 g) were purchased from The First Affiliated Hospital of Nanchang University Animal Experimental Center. They fasted overnight before the experiment. The rats underwent peritoneal injection of 25 μg/kg Dex (Orion Pharma), 10 mg/kg SB239063 (p38MAPK inhibitor; Sigma‐Aldrich Chemical Company), 250 μg/kg Atipamezole (α2AR antagonist; Sigma‐Aldrich), and 11.49 mg/kg Gypenoside (GP; NF‐κB inhibitor; ZL150307100, Nanjing Zelang Medical Technology Co., Ltd.) 30 minutes before intestinal I/R modelling. At 30 minutes after injection, the intestinal I/R model was constructed. The rats in all experimental groups were anesthetized with pentobarbital sodium (390 mg) and phenytoin sodium (50 mg/mL),^15^ and the abdomen was entered via midline incision. The superior mesenteric artery (SMA) was identified and dissected by a blunt cutter. A microvascular clamp was placed at the root of the SMA to stop the blood flow completely for 60 minutes and then was released to form a reperfusion injury. At the end of the experiment, all animals were euthanized by an anaesthesia overdose. Blood samples and intestinal biopsies were then collected. The right upper lobes of the lungs were fixed with 4% paraformaldehyde and prepared for further pathological study to analyse the histomorphology and ultrastructural changes. The right middle lobes of the lungs were used to determine the wet/dry weight ratio of the lung. The right lower lobes of the lung tissues were crushed, dissolved and separated for Western blot analysis. The remaining lung tissue fluid was put immediately into liquid nitrogen for cryopreservation.

The rats either received no SMA occlusion procedures (intestinal I/R injury) as sham group or received intestinal I/R injury as experimental groups in four groups: intestinal I/R alone and intestinal I/R injury with reperfusion followed by survival for 6, 12, 24 or 48 hours. that were injected separately with four different adenoviruses including adenoviruses‐short hairpin RNA (Ad‐sh)‐Cav‐1, Ad‐sh‐negative control (NC), Ad‐overexpression (oe)‐Cav‐1 and Ad‐oe‐NC, all purchased from Shanghai GeneChem Co., Ltd., at four days before intestinal I/R application. The rats were injected intraperitoneally with 0.3 mL adenovirus at a dose of 1 × 10^9^ plaque‐forming units (PFU) 4 days before intestinal I/R modelling. The final two experimental groups were treated with both Dex (30 minutes before intestinal I/R) and Ad‐sh‐Cav‐1 (four days before intestinal I/R), or Dex and Ad‐sh‐NC. The survival number of rats in each group was different, with details shown in Table S1. Four‐to‐five rats from each group were collected for follow‐up experiments. Another 20 rats following intestinal I/R modelling were euthanized at 6, 12, 24 and 48 hours after modelling, and their lung tissues were collected, with 3‐5 rats in each group.

### 2.2 Wet‐to‐dry lung weight ratio

The extra lung tissues were carefully removed after harvesting the right middle lobe of the lung. The right middle lobe was then rinsed with physiological saline, blotted on filter paper and weighed when wet. The lobe was then dried at 60°C for 48 hours, and the residuum was also weighed. The ratio of wet to dry weight and lung water content were calculated by using the equation [(lung wet weight‐lung dry weight)/lung wet weight].^16^

### 2.3 Haematoxylin‐eosin (H&E) staining

The rat intestines and lungs were collected, fixed and embedded in paraffin. The samples were cut into 4‐μm‐thick slices, dewaxed with xylene, and with toluene for 5 minutes, and rehydrated with 100% ethanol for 2 minutes, 95% ethanol for 1 minute, 80% ethanol for 1 minute, 75% ethanol for 1 minute and distilled water for 2 minutes. All samples were then stained with haematoxylin for 5 minutes and differentiated with dihydrochloric acid and ethanol for 30 seconds. The slides were soaked in tap water for 15 minutes (or 50°C warm water for 5 minutes) and then placed into eosin solution for 2 minutes. An additional three steps of routine dehydration, clearing, and neutral resin sealing were performed. To remove all traces of water, samples were rinsed in several alcohol baths to complete the clearing process: 95% ethanol for 1 minute, 95% ethanol for 1 minute, 100% ethanol for 1 minute, 100% ethanol for 1 minute, toluene carbonic acid (3:1) for 1 minute, toluene for 1 minute, and xylene for 1 minute. At last, samples were mounted and evaluated via an inverted microscope (Olympus Corporation).

### 2.4 Evaluation of intestinal and lung injuries

The five‐point Chiu score was used to evaluate the degree of the intestinal injury according to the changes of intestinal mucosal villi and glands.^17^ The degrees of injury were Grade 0: normal mucosal villi; grade 1: development of a subepithelial space, often accompanied with capillary congestion; grade 2: prolongation of subepithelial space and moderate elevation of lamina propria epithelium; grade 3: a large number of epithelial cells rising along the side of the villus, possibly with a small amount of exfoliation; grade 4: lamina propria villi shedding, telangiectasia, lamina propria cells increase; grade 5: degradation and disintegration of the lamina propria, haemorrhage, and ulceration.

The injury of the lung specimens was evaluated on a scale from 0 (best) to 3 (worst) as follows: grade 0: normal structure of lung tissues; grade 1: mild alveolar wall oedema; a small amount of inflammatory cell infiltration in the interstitium; a small amount of bleeding in mesenchymal and alveolar cavity; grade 2: moderate alveolar wall oedema; profuse inflammatory cell infiltration in mesenchymal and alveolar cavity; capillary congestion and haemorrhage; grade 3: extensive alveolar and interstitial oedema; a large number of infiltrating inflammatory cells occurred in most alveoli and interstitium and severe alveolar haemorrhage.

At least 5 visual fields were randomly selected from the samples of each rat and were scored and evaluated independently by two pathologists.

### 2.5 Enzyme‐linked immunosorbent assay (ELISA)

Interleukin (IL)‐6 ELISA kit (PI328, Beyotime, Shanghai, China) was used to measure the IL‐6 level of the lung homogenates. The optical density (OD) values of the testing samples were measured, and the level of IL‐6 in the sample was calculated from the standard curve generated by the standard samples offered within the ELISA kit. Tumour necrosis factor‐α (TNF‐α) (PT516, Beyotime) and IL‐1β (PI303, Beyotime) kit testing was also conducted in this experiment. Furthermore, the levels of intestinal mucosal injury markers (i‐FABP) and diaminoxidase (DAO) in portal vein blood were determined by an ELISA kit supplied by Wuhan USCN Business, according to the manufacturer's instructions.

### 2.6 Terminal deoxynucleotidyl transferase‐mediated dUTP‐biotin nick end labelling (TUNEL) staining

Apoptosis of hepatocytes was determined using the TUNEL staining kit (G3250, Promega) according to the manufacturer's instructions. All samples were then photographed and observed under a microscope (BX51, Olympus).^18^

### 2.7 Reverse transcription quantitative polymerase chain reaction (RT‐qPCR)

The total RNA was extracted from tissues and cells with TRIzol reagents (15596026, Invitrogen Inc.) and then reverse transcribed into complementary DNA (cDNA) using the Script One‐Step RT‐PCR kit (Takara Bio Inc). RT‐qPCR was then conducted using SYBR Premix EX Taq kit (RR420A, Takara) on an ABI 7500 instrument (Applied Biosystems). All investigations involved at least 3 wells, each repeated in triplicate. The primer sequences are shown in Table S2. The fold changes were calculated using relative quantification (the 2^‐ΔΔCt^ method) with glyceraldehyde‐3‐phosphate dehydrogenase (GAPDH) serving as loading control.

### 2.8 Western blot analysis

The total protein was extracted from tissues, and the protein concentration was determined by a bicinchoninic acid (BCA) kit (Thermo Fisher Scientific Inc.). Then, 30 μg of total protein was used for polyacrylamide gel electrophoresis and transferred onto polyvinylidene fluoride (PVDF) membranes (Amersham, Pharmacia Biotech). The PVDF membrane was blocked in 5% skimmed milk at room temperature for 1 hours and incubated overnight at 4°C with the diluted primary antibodies: rabbit anti‐α_2A_‐AR (1:1000, 14266‐1‐AP, Proteintech ProteinTech Group), rabbit anti‐Cav‐1 (1:1000, ab32577, Abcam Inc.), rabbit anti‐p38 (1:1000, ab31828, Abcam), rabbit anti‐phosphorylated (p)‐p38 (1:1000, ab4822, Abcam), rabbit anti‐NF‐κBp65 (1:1000, ab16502, Abcam), rabbit anti‐NF‐κB‐p‐p65 (1:1000, ab86299, Abcam) and rabbit anti‐Actin (1:1000, ab179467, Abcam). The next day, the membrane was washed three times with PBS containing 0.1% Tween‐20 (PBST) for 10 minutes each time. Secondary anti‐IgG antibodies were added to the membrane and incubated at room temperature for 1 hours. The membrane was repeatedly washed in PBST as above. The relative expression of the protein bands was detected and evaluated by Image‐Pro Plus 6.0 software (Media Cybernetics). Each test was conducted in triplicate for accuracy.

### 2.9 Statistical analysis

All statistical data were expressed as mean ± standard deviation and processed by SPSS 21.0 statistical software (IBM Corp.). The comparisons between groups were analysed by one‐way analysis of variance (ANOVA) with Tukey's post‐test. *P* < .05 indicates that the difference is statistically significant.

## 3 RESULTS

### 3.1 Intestinal I/R induces lung injury in rats

**FIGURE 1:**
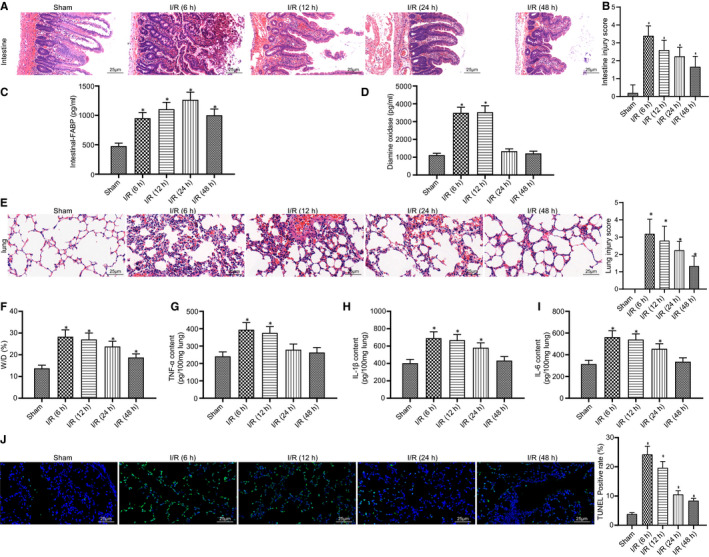
The relationship between the dynamic changes of intestinal/pulmonary inflammation and the elapsed times after reperfusion during intestinal I/R injury. A, Representative images of pathological changes in intestinal tissues with various elapsed time. (400×, scale bar = 25 μm). B, Intestinal injury scores with and without I/R injury in different reperfusion time points. C, i‐FABP expression in portal vein blood varied with times. D, DAO expression in portal vein blood varied with times. E, Injury scoring and histopathological studies of lung tissues (400×, scale bar = 25 μm). F, The wet‐to‐dry weight ratio of the lung tissues as the measurement of the oedema. G, ELISA detection of TNF‐α expression in lung tissues. H, ELISA detection of IL‐1β expression in lung tissues. I, ELISA detection of IL‐6 expression in lung tissues. J, TUNEL assay detection for cell apoptosis in lung tissues (400×, scale bar = 25 μm). *P < .05 vs. sham group. Data among multiple groups were analysed by one‐way ANOVA with Tukey's post hoc test

Initially, we constructed an intestinal I/R injury model and observed the changes of inflammation and the degree of lung injury. H&E staining analysis showed a severe filling of capillaries and destruction of intestinal villi, but these conditions were gradually alleviated with increasing time after reperfusion compared to the sham group (Figure 1A). The highest intestinal injury score was also evaluated at the 6 hours after reperfusion (Figure 1B) (all *P* < .05). Although the highest level of i‐FABP in portal blood was observed at 24 hours, i‐FABP expression was up‐regulated at 6 hours compared to the sham group (Figure 1C) (all *P* < .05). The activity of DAO, another inflammatory indicator, increased at 6 and 12 hours after reperfusion and decreased at 24 and 48 hours (Figure 1D) (all *P* < .05). Analysis on the lung tissues of rats using H&E staining suggested that after reperfusion for 6 hours, a series of acute inflammatory events occurred, indicated by alveolar wall thickening, pulmonary interstitial and alveolar haemorrhage and oedema, and infiltration of abundant immune cells. However, these pathological findings began to decline at 12 hours after the reperfusion (Figure 1E). Therefore, the peak lung injury score occurred at 6 hours after reperfusion (Figure 1E) (all *P* < .05). The wet‐to‐dry lung weight ratio indicated that the most severe pulmonary oedema with fluid accumulation occurred at 6 hours and slowly resolved at 24 hours (Figure 1F) (all *P* < .05). Additionally, the expression of the major inflammatory factors (TNF‐α, IL‐1β and IL‐6) also peaked at 6 hours after reperfusion, which was normalized at 48 hours (Figure 1G‐I) (all *P* < .05). The results of TUNEL staining demonstrated that cell apoptosis in rat lung tissues was the most severe at 6 hours and gradually alleviated with increasing time after reperfusion (Figure 1J) (all *P* < .05). All the above results showed that the lung injury induced by intestinal I/R was a dynamic process, with 6 hours after reperfusion being a critical time point that was selected for the subsequent experiments.

### 3.2 Dex promotes Cav‐1 expression via α_2A_‐AR activation and mitigates intestinal I/R‐induced lung injury

Dex acts as a α_2A_‐AR agonist with high potency and selectivity, thereby reducing the release of inflammatory factors and cytokines. Some previous literature has shown that Dex can alleviate intestinal I/R injury.^15^ In this study, we further studied the protective effects of Dex against lung injury due to intestinal I/R injury by mediating the expression of Cal‐1 and α_2A_‐AR.

**FIGURE 2:**
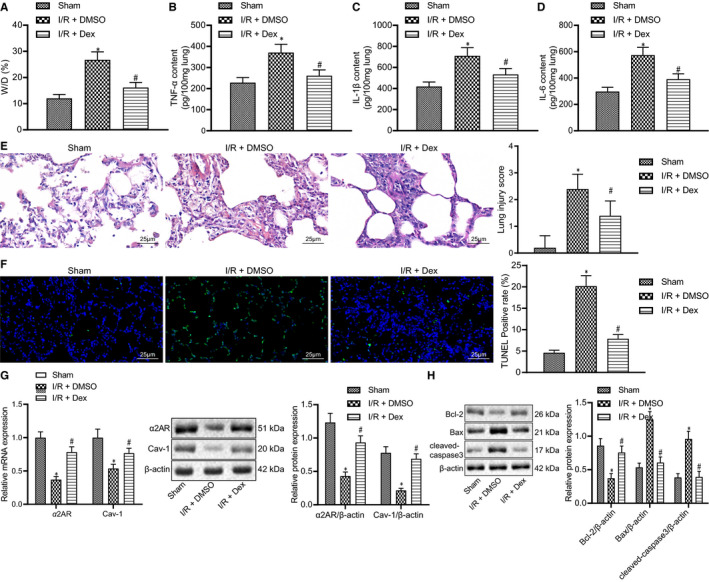
Up‐regulation of Cav‐1 by Dex through α_2A_‐AR alleviates intestinal I/R‐induced lung injury. A, The wet‐to‐dry weight ratio of lung tissues with DMSO or Dex treatment prior to intestinal I/R injury. B, ELISA detection of TNF‐α expression in rat lung tissues with DMSO or Dex treatment prior to intestinal I/R injury. C, ELISA detection of IL‐1β expression in rat lung tissues with DMSO or Dex treatment prior to intestinal I/R injury. D, ELISA detection of IL‐6 expression in rat lung tissues with DMSO or Dex treatment prior to intestinal I/R injury. E, Histopathological examination of rat lung tissues with DMSO or Dex treatment prior to intestinal I/R injury by H&E staining (400×, scale bar = 25 μm). F, Apoptosis of rat lung tissues with DMSO or Dex treatment prior to intestinal I/R injury by TUNEL staining (400×, scale bar = 25 μm). G, RT‐qPCR and Western blot analysis of α_2A_‐AR and Cav‐1 expressions in rat lung tissues with DMSO or Dex treatment prior to intestinal I/R injury. H, Western blot analysis of cleaved caspase‐3, Bax and Bcl‐2 proteins in rat lung tissues with DMSO or Dex treatment prior to intestinal I/R injury. *P < .05 vs sham group, ^#^ *P* < .05 vs I/R+ DMSO group. Data among multiple groups were analysed by one‐way ANOVA with Tukey's post hoc test

Our results showed that the wet‐to‐dry lung weight ratio in the I/R group was higher than that of the sham group. On the other hand, the ratio was at the level of the sham group after Dex pretreatment and showed only a minor oedema as opposed to the I/R group (Figure 2A) (all *P* < .05). The levels of TNF‐α, IL‐1β and IL‐6 were elevated in lung tissues of rats with intestinal I/R injury, along with increased mortality rates (Table S1), but were normalized by the pre‐injection of Dex to the control levels along with decreased mortality rates (Table S1) (Figure 2B‐D) (all *P* < .05). The histology studies illustrated that the structure of lung tissues in the sham group was normal, with only a small amount of exudation in alveolar cavity and no obvious congestion with haemorrhage in capillaries. However, the I/R group was accompanied by apparent destruction of alveolar structure, thickened pulmonary interstitium, alveolar haemorrhage and oedema and accumulated inflammatory cells in the injured sites. The Dex group showed only faint signs of destruction and inflammation, characterized by less exudation of the pulmonary interstitium, decreased alveolar cavity volumes, few disrupted alveoli and inflammatory cell infiltration and a slightly thickened alveolar septum (Figure 2E) (all *P* < .05). Additionally, the degree of apoptosis in lung tissues was lower in the Dex group than that in the I/R group (Figure 2F) (all *P* < .05). Western blot analysis results showed that the expression of cleaved caspase‐3 and Bax proteins was increased in lung tissues of intestinal I/R rats compared with the sham group, while that of Bcl‐2 protein was decreased. However, Dex treatment brought about a contrary trend (Figure 2H). Thus, pre‐injection of Dex has a certain protective effect on lung injury induced by intestinal I/R.

We next further explored the mechanism of the protective effects of Dex on intestinal I/R‐initiated lung injury. Dex has been reported to up‐regulate the expression of Cav‐1 in the lung tissues of septic rats and to inhibit inflammation processes.^12^ The results of RT‐qPCR and Western blot analysis showed that the mRNA and protein expression of Cav‐1 and α_2A_‐AR was reduced in lung tissues of rats with intestinal I/R injury, while an opposite result was noted in lung tissues of rats with Dex pre‐injection, indicating a correlation of α_2A_‐AR with Cav‐1 associated inflammation (Figure 2G) (all *P* < .05). These results also suggested that pre‐injection of Dex attenuated lung injury through activation of α_2A_‐AR and up‐regulation of Cav‐1.

### 3.3 Dex impairs lung injury induced by intestinal I/R by facilitating α_2A_‐AR‐dependent p38MAPK/NF‐κB pathway inactivation

**FIGURE 3:**
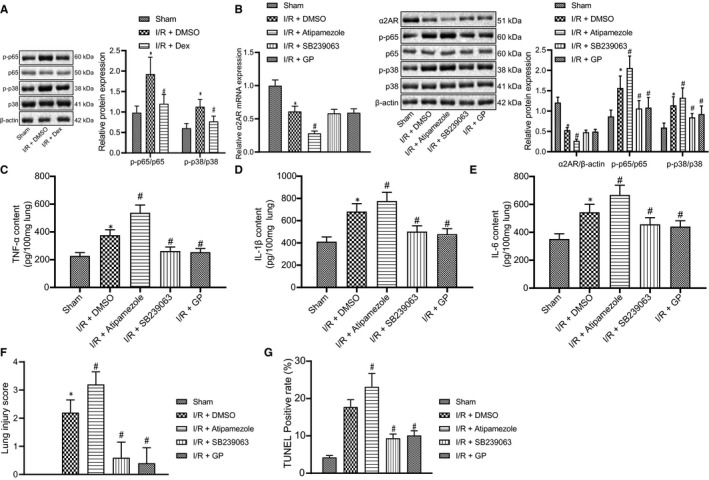
Dex reduces lung injury following intestinal I/R *via* α2A‐AR‐dependent p38MAPK/NF‐κB pathway inactivation. A, Western blot analysis of α_2A_‐AR, p38, p‐p38, p‐p65 and p65 in rat lung tissues with DMSO or Dex treatment prior to intestinal I/R injury. B, RT‐qPCR of α_2A_‐AR mRNA expression and Western blot analysis of α_2A_‐AR, p38, p‐p38, p‐p65 and p65 in rat lung tissues with injections of Atipamezole, SB239063 and GP inhibitors prior to intestinal I/R injury. C, ELISA detection of TNF‐α expression in rat lung tissues with injections of Atipamezole, SB239063 and GP inhibitors prior to intestinal I/R injury. D, ELISA detection of IL‐1β expression in rat lung tissues with injections of Atipamezole, SB239063 and GP inhibitors prior to intestinal I/R injury. E, ELISA detection of IL‐6 expression in rat lung tissues with injections of Atipamezole, SB239063 and GP inhibitors prior to intestinal I/R injury. F, Lung injury score of rat lung tissues with injections of Atipamezole, SB239063 and GP inhibitors prior to intestinal I/R injury. G, Apoptosis rate of rat lung tissues with injections of Atipamezole, SB239063 and GP inhibitors prior to intestinal I/R injury, detected by TUNEL staining. *P < .05 vs sham group, ^#^ *P* < .05 vs I/R +DMSO group. Data among multiple groups were analysed by one‐way ANOVA with Tukey's post hoc test

Lung injury has been shown to initiate activation of the p38MAPK pathway and result in the increased secretion of cytokines such as MAPK and NF‐κB. Meanwhile, inhibition of the p38MAPK/NF‐κB pathway has protective effects on tissues and organs in the intestinal I/R model.^19^ Our previous study found that Dex had an anti‐apoptotic effect on intestinal I/R injury.^15^ In this experiment, we intended to examine the effects of Dex on the expression of α_2A_‐AR, MAPK and NF‐κB after injury. The results of Western blot analysis demonstrated higher phosphorylation levels of p56 and p38 in the I/R group than that in the sham group, but Dex pretreatment reversed this trend (Figure 3A) (all *P* < 05). An opposite result was found for α_2A_‐AR expression. Thus, the protective effect of Dex on intestinal I/R‐induced lung injury may be closely related to α_2A_‐AR/p38MAPK/NF‐κB pathway.

Western blot analysis further indicated that compared to the I/R group, the phosphorylation level of p56 and p‐p38/p38 ratio was increased in the Atipamezole group along with decreased α_2A_‐AR mRNA and protein expression. Conversely, the phosphorylation level of p56 and p‐p38/p38 ratio was diminished in the SB239063 group, and phosphorylation level of p56 was decreased in the GP group (Figure 3B) (all *P* < .05), thus indicating an inverse correlation between α_2A_‐AR and p38MAPK, NF‐κB. The results also demonstrated that the levels of IL‐6, IL‐1β and TNF‐α were raised in the Atipamezole group, but declined both in the SB239063 and GP groups (Figure 3C‐E) (all *P* < .05 vs sham). This reveals the role and relationship of α_2A_‐AR in the inflammation related p38MAPK/NF‐κB pathway, as also confirmed by results of the pathology. In particular, the degree of pulmonary interstitial and alveolar exudation was lower in the SB239063 and GP groups, which showed little structural disruption and few inflammatory signs. However, Atipamezole treatment aggravated intestinal I/R injury, showing an increment of alveoli destruction, haemorrhage and oedema (Figure S1A; Figure 3F) (all *P* < .05). The outcomes of TUNEL staining further affirmed the mechanism of the α_2A_‐AR/p38MAPK/NF‐κB pathway, with an escalation of apoptosis in the Atipamezole group, but reduction in the SB239063 and GP groups, because of their inhibition of p38MAPK and NF‐κB (Figure S2A; Figure 3G) (all *P* < .05). Additionally, the mortality rate of the Atipamezole group increased, while that of the SB239063 and GP groups decreased, when compared with the I/R group (Table S1). We concluded that lung injury in rats can be affected by the promotion of α_2A_‐AR signalling and consequent inhibition of the expression of p38MAPK and NF‐κB.

### 3.4 Dex impairs lung injury induced by intestinal I/R by facilitating Cav‐1‐dependent p38MAPK/NF‐κB pathway inactivation

Down‐regulation of Cav‐1 can enhance the phosphorylation levels of p38 and the expression of NF‐κB and exacerbate LPS‐induced acute lung injury.^13^ The up‐regulation of Cav‐1 can regulate the production of pro‐inflammatory cytokines TNF‐α and IL‐6 induced by LPS in mouse peritoneal and alveolar macrophages *via* the p38MAPK pathway.^14^ Therefore, in our study, we also analysed the effects of Dex on pulmonary inflammatory injury by promoting Cav‐1 to inhibit the activation of p38 and NF‐κB in lung injury.

**FIGURE 4:**
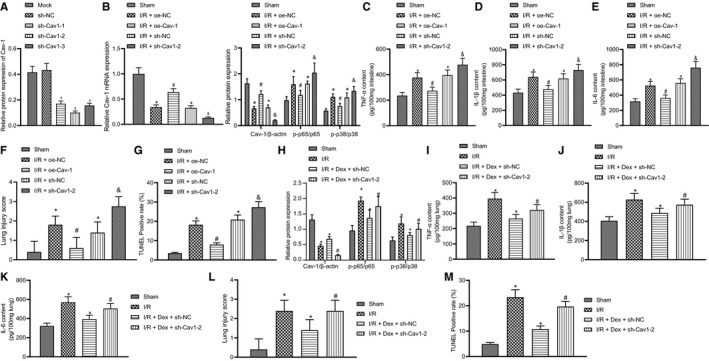
Dex reduces lung injury following intestinal I/R *via* Cav‐1‐dependent p38MAPK/NF‐κB pathway inactivation. A, Comparison of the down‐regulation for Cav‐1 protein using three anti‐Cav‐1 shRNA constructs. *P < .05 compared with the mock group. Pre‐injections were applied four days before intestinal I/R injury. B, RT‐qPCR of Cav‐1 mRNA expression and Western blot analysis of Cav‐1, p38, p‐p38, p‐p65 and p65 expression in rat lung tissues with Cav‐1 overexpression or knockdown before intestinal I/R injury. C, ELISA detection of TNF‐α expression in rat lung tissues with Cav‐1 overexpression or knockdown before intestinal I/R injury. D, ELISA detection of IL‐1β expression in rat lung tissues with Cav‐1 overexpression or knockdown before intestinal I/R injury. E, ELISA detection of IL‐6 expression in rat lung tissues with Cav‐1 overexpression or knockdown before intestinal I/R injury. F, Lung injury score of rat lung tissues with Cav‐1 overexpression or knockdown before intestinal I/R injury. G, Apoptosis rate of rat lung tissues with Cav‐1 overexpression or knockdown before intestinal I/R injury. *P < .05 vs sham group, ^#^ *P* < .05 vs I/R + oe‐NC group. H, Western blot analysis of Cav‐1, p38, p‐p38, p‐p65 and p65 in rat lung tissues upon Dex treatment and/or Cav‐1 knockdown prior to intestinal I/R injury. I, ELISA detection of TNF‐α expression in rat lung tissues upon Dex treatment and/or Cav‐1 knockdown prior to intestinal I/R injury. J, ELISA detection of IL‐1βexpression in rat lung tissues upon Dex treatment and/or Cav‐1 knockdown prior to intestinal I/R injury. K, ELISA detection of IL‐6 expression in rat lung tissues upon Dex treatment and/or Cav‐1 knockdown prior to intestinal I/R injury. L, Lung injury score of rat lung tissues upon Dex treatment and/or Cav‐1 knockdown prior to intestinal I/R injury. M, Apoptosis rate of rat lung tissues upon Dex treatment and/or Cav‐1 knockdown prior to intestinal I/R injury, detected by TUNEL staining. *P < .05 vs sham group, ^#^ *P* < .05 vs I/R + Dex +sh‐NC group. Data among multiple groups were analysed by one‐way ANOVA with Tukey's post hoc test

Western blot analysis results found that the sh‐Cav‐1‐2 out of three types of Ad‐sh‐Cav‐1 viruses was the most effective and applicable (Figure S3A; Figure 4A). In addition, Western blot analysis further illustrated an upturn of Cav‐1 mRNA and protein expression and a downturn of phosphorylation levels of p56 and p38 in the Ad‐oe‐Cav‐1 group, compared to the Ad‐oe‐NC group. However, the Ad‐sh‐Cav‐1‐2 group had completely opposite outcomes, as shown by decreased mRNA and protein expression of Cav‐1 and enhanced phosphorylation levels of p56 and p38 (Figure 4B). The results of ELISA showed that the expression of IL‐6, IL‐1 and TNF‐α was lower in lung tissues in Ad‐oe‐Cav‐1‐2 rats than that in Ad‐oe‐NC rats (all *P* < .05), suggesting that up‐regulation of Cav‐1 blocks the inflammation process. In comparison with the Ad‐sh‐NC group, the expression of IL‐6, IL‐1 and TNF‐α was increased in the Ad‐sh‐Cav‐1‐2 group (all *P* < .05) (Figure 4C‐E). According to the results of H&E staining, the exudation of pulmonary interstitium and alveolar cavity was attenuated in the Ad‐oe‐Cal‐2 group as compared to the Ad‐oe‐NC group. However, the Ad‐sh‐Cav‐1‐2 rats showed aggravation of the lung injury caused by intestinal I/R. The exudation of pulmonary interstitium and alveolar cavity was increased in these animals, in which the alveolar structure was destroyed and the alveolar wall was thickened. Meanwhile, there were pulmonary interstitial/alveolar haemorrhaging along with oedema and elevated infiltration of inflammatory cells (Figure S1B; Figure 4F). The results of TUNEL assay indicated that the Ad‐oe‐Cav‐1‐2 group had restrained cell death but the Ad‐sh‐Cav‐1‐2 group showed aggravation of the apoptotic processes after intestinal I/R injury (Figure S2B; Figure 4G). Additionally, relative to the I/R + oe‐NC group, the mortality rate of the I/R + sh‐Cav‐1 group increased while that of the I/R+ oe‐Cav‐1 group decreased (Table S1). In summary, Ad‐oe‐Cav‐1‐2 treatment can alleviate pulmonary inflammation induced by intestinal I/R by suppressing the p38MAPK/NF‐κB pathway.

Next, we further verified whether Dex can alleviate pulmonary inflammatory injury by promoting Cav‐1 The results of Western blot analysis showed higher phosphorylation levels of p56 and p38 in the Ad‐sh‐Cav‐1 group than in the Ad‐sh‐NC group (Figure S3B; Figure 4H). The expression of IL‐6, IL‐1 and TNF‐α in lung tissues showed the same results, with increased inflammation in the Ad‐sh‐Cav‐1‐2 group (Figure 4I‐K) (all *P* < .05). Thus, compared with Ad‐sh‐NC rats, the lung tissues in the Ad‐sh‐Cav‐1‐2 group had aggravated injury caused by intestinal I/R. The alveolar structure in these animals was destroyed and the alveolar wall thickened. Meantime, haemorrhage and oedema were present both in the pulmonary interstitium and alveoli, with excessive infiltration of immune cell (Figure S1CC; Figure 4L) (all *P* < .05). TUNEL staining showed that the number of apoptotic cells in Ad‐sh‐Cav‐1‐2 treated rats was higher than that in Ad‐sh‐NC treated rats (Figure S2C; Figure 4M) (all *P* < .05). Furthermore, the mortality rate was much higher in the I/R+ Dex + sh‐Cav‐1 group than that in the I/R+ Dex + sh‐NC group (Table S1). These results suggested that Dex could alleviate pulmonary inflammation by promoting Cav‐1 to inhibit the activation of p38 and NF‐κB in intestinal I/R‐initiated lung injury.

### 3.5 Dex reduces inflammatory response of lung injury induced by intestinal I/R injury by regulating the α_2A_‐AR/Cav‐1/p38MAPK/NF‐κB axis

**FIGURE 5:**
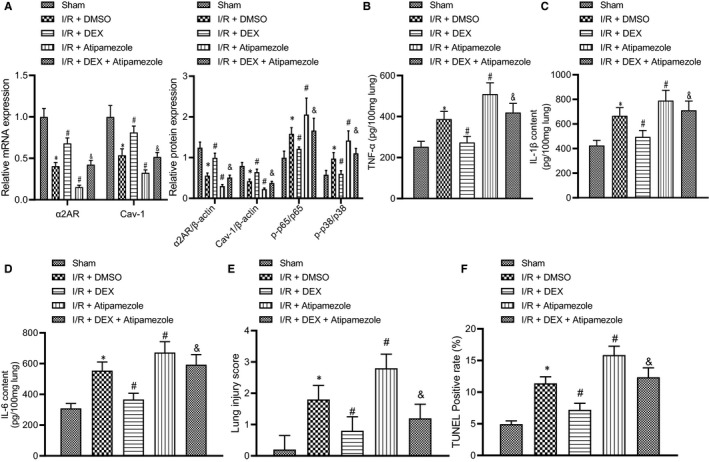
Dex suppresses inflammatory response of lung injury induced by intestinal I/R injury by regulating the α2A‐AR/Cav‐1/p38MAPK/NF‐κB axis. A, RT‐qPCR of Cav‐1 and α_2A_‐AR mRNA expression and Western blot analysis of α_2A_‐AR, Cav‐1, p38, p‐p38, p‐p65 and p65 in rat lung tissues after combination treatment of Dex and Atipamezole prior to intestinal I/R injury. B, ELISA detection of TNF‐α expression in rat lung tissues after combination treatment of Dex and Atipamezole prior to intestinal I/R injury. C, ELISA detection of IL‐1β expression in rat lung tissues after combination treatment of Dex and Atipamezole prior to intestinal I/R injury. D, ELISA detection of IL‐6 expression in rat lung tissues after combination treatment of Dex and Atipamezole prior to intestinal I/R injury. E, Lung injury score of rat lung tissues after combination treatment of Dex and Atipamezole prior to intestinal I/R injury by H&E staining. F, Apoptosis rate of rat lung tissues after combination treatment of Dex and Atipamezole prior to intestinal I/R injury, detected by TUNEL staining. *P < .05 vs sham group, ^#^ *P* < .05 vs I/R+ DMSO group, ^&^ *P* < .05 vs I/R+ Atipamezole group

Finally, we further elucidated whether Dex attenuates the lung injury induced by intestinal I/R injury by regulating the α2A‐AR/Cav‐1/p38MAPK/NF‐κB axis. The results of RT‐qPCR and Western blot analysis presented reduced mRNA and protein expression of α_2A_‐AR and Cav‐1 and enhanced phosphorylation levels of p56 and p38 in lung tissues of rats treated with Atipamezole (Figure S3C; Figure 5A) (all *P* < .05). On the contrary, Dex enhanced the mRNA and protein expression of α_2A_‐AR and Cav‐1 and suppressed the phosphorylation levels of p56 and p38 Conjoint treatment with Dex and Atipamezole abolished the effect of Dex alone (Figure 5A) (all *P* < .05). ELISA data revealed that the secretion of TNF‐α, IL‐1β and IL‐6 was mitigated with the treatment of Dex in conjunction with Atipamezole, suggesting the preventive effects of Dex (Figure 5B‐D) (all *P* < .05). H&E staining analysis indicated that co‐treatment of Atipamezole with Dex demonstrated the efficiency and protective effects of Dex against the Atipamezole and lung injury (Figure S1D; Figure 5E). Apoptosis studies of Dex plus Atipamezole group showed a moderate reduction of cell death due to the aggravation of Atipamezole alone on the injury. Nevertheless, Dex eased the inhibition of Atipamezole on α_2A_‐AR and the activation of α_2A_‐AR/Cav‐1/p38MAPK/NF‐κB pathway (Figure S2D; Figure 5F) (all *P* < .05), with the decreased mortality rate observed (Table S1). In conclusion, Dex reduced pulmonary inflammatory response even in the face of intestinal I/R injury and Atipamezole treatment.

## 4 DISCUSSION

**FIGURE 6:**
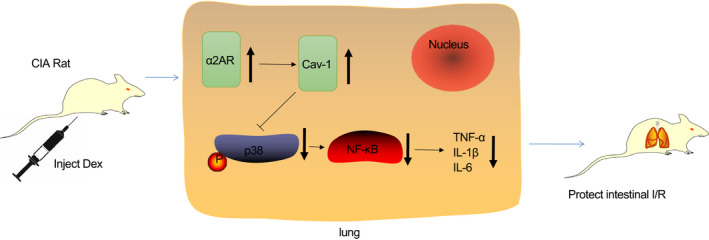
A graphic abstract of the molecular mechanisms underlying protection by Dex against lung injury following intestinal I/R injury. Dex administration can up‐regulate the expression of Cav‐1 by promoting α2AR, thereby inhibiting the activation of p38 and NF‐κB in lung injury caused by intestinal I/R and alleviating lung inflammation injury

The intestine is one of the most sensitive organs to ischaemia. Intestinal I/R can not only cause damage to the intestine itself, but also cause damage to many remote organs, even leading to multiple organ dysfunction syndromes.^20^ Lung injury is one of the common organs involved in intestinal I/R injury. In the worst case, intestinal I/R injury can even cause a life‐threatening respiratory distress syndrome.^17^ Although a large number of studies have been carried out on lung injury caused by intestinal I/R in recent years, its exact pathogenesis has not been fully elucidated, and there is still a lack of effective prevention and treatment measures. In our present study, we established an intestinal I/R injury rat model to investigate the effect of Dex on intestinal I/R injury‐induced ALI with the involvement of the α2AR/Cav‐1/p38MAPK/NF‐κB axis. Collectively, our results showed that Dex can up‐regulate the expression of Cav‐1 by promoting α2AR, thereby inhibiting the activation of p38 and NF‐κB in lung injury caused by intestinal I/R injury (Figure 6).

Our research confirmed that intestinal I/R injury in rats can cause significant lung injury. When the reperfusion time was 6 hours, the degree of lung injury was the worst, which was consistent with the time course of changes of inflammatory factors such as increased levels of TNF‐α, IL‐1β and IL‐6. Intestinal I/R results in the destruction of the intestinal mucosal barrier and structure and the release of large amounts of endotoxin into the blood, which leads to uncontrolled release of cytokines and inflammatory mediators throughout the body.^21^ We also found that the Dex treatment can reduce the pathological damage of lung tissues and the infiltration of inflammatory cells upon intestinal I/R injury. Dex is a relatively new type of highly potent and selective α2AR agonist, which can reduce the release of inflammatory factors and cytokines. Present findings of the effect of Dex on intestinal I/R injury confirm a previous report.^15^ Moreover, our experimental results also show that with Dex treatment, the expression of Cav‐1 and α2AR in the lung tissues of rats increased significantly. This indicates that the pre‐injection of Dex can alleviate lung injury caused by intestinal I/R by up‐regulating the expression of α2AR and Cav‐1. Li et al previously found Dex can increase the expression of Cav‐1 in lung tissues of rats with sepsis and improve the short‐term outcome,^12^ which was partially in line with our findings. Furthermore, according to another previous study, Dex can up‐regulate the expression of Cav‐1 in lung tissues of sepsis rats and help to inhibit inflammation.^12^ Down‐regulation of Cav‐1, activation of p‐p38 and NF‐κB increased the severity of LPS‐induced acute lung injury.^13^ Then, the specific mechanisms in this pathway were further investigated in our work.

As mentioned above, the p38MAPK/NF‐κB pathway plays an important role in the model of intestinal I/R lung injury. During the process of intestinal I/R lung injury, p38MAPK is activated, and the expression of MAPK, NF‐κB and other inflammatory cell factors is increased.^19^ MAPK pathway is one of the important inflammatory pathways in cells, and a large number of studies have proved that the MAPK pathway plays an important role in I/R injury of various organs such as heart and intestine.^22^, ^23^ As one of the main members of the MAPK family, p38 plays an important role in the occurrence and development of ALI.^24^, ^25^ To confirm whether Dex affects the expression of p38MAPK/NF‐κB during intestinal I/R lung injury, we tested the lung tissues of rats in different treatment groups. Our results supported the conclusion that pre‐injection of Dex to relieve ALI caused by intestinal I/R injury involves p38MAPK/NF‐κB pathway. In LPS‐induced ALI, inhibiting the expression of Cav‐1 can promote the activation of p‐p38 and NF‐κB.^13^ At the same time, Cav‐1 can regulate the production of the pro‐inflammatory cytokines (TNF‐α and IL‐6) induced by LPS in mouse peritoneal and alveolar macrophages by the MKK3/p38MAPK pathway.^14^ Therefore, we speculate that Dex may alleviate intestinal I/R injury‐induced ALI by promoting Cav‐1 through the activation of p38 and NF‐κB.

After confirming that Dex can regulate Cav‐1/p38MAPK/NF‐κB pathway to inhibit the inflammatory response during intestinal I/R lung injury, we used the α2AR antagonist together with Dex to treat the intestinal I/R lung injury model. As expected, the expression of Cav‐1 decreased after α2AR was blocked, indicating that Cav‐1 is a downstream signalling factor of α2AR. Thus, Dex can regulate the α2AR/Cav‐1/p38MAPK/NF‐κB pathway to play a protective role in intestinal I/R lung injury model.

Although we have established that Dex can inhibit the inflammatory response by regulating the p38MAPK/NF‐κB pathway during intestinal I/R lung injury, we have not investigated other potentially relevant pathways. For example, an excessive oxidative stress response is one of the important mechanisms in intestinal I/R lung injury,^26^ but we did not yet explore whether Dex has any effect on oxidative stress response in this process. Besides, in this work, we mainly focused on the effects of intestinal I/R injury on lung inflammation and apoptosis, but did not pay attention to the changes of ROS due to the lack of sample size, which is also the limitation of our study. In future studies, we will pay more attention to this issue.

## 5 CONCLUSION

In conclusion, Dex can up‐regulate the expression of Cav‐1 by promoting α2AR activation, thereby inhibiting the activation of p38 and NF‐κB in lung injury caused by intestinal I/R and alleviating lung inflammation injury.

## CONFLICT OF INTEREST

The authors declare no competing financial interests.

## AUTHOR CONTRIBUTIONS

**Lin Xu:** Conceptualization (equal); Resources (equal). **Taiyuan Li:** Formal analysis (equal); Investigation (equal). **Qiuhong Chen:** Supervision (equal); Writing‐original draft (equal). **Zhen Liu:** Project administration (equal); Writing‐review & editing (equal). **Yuesheng Chen:** Data curation (equal); Writing‐review & editing (equal). **Kai Hu** **:** Visualization (equal); Writing‐original draft (equal). **Xuekang Zhang:** Formal analysis (equal); Methodology (equal).

## Supporting information

Figure S1

Figure S2

Figure S3

Table S1‐S2

## Data Availability

Research data are not shared.
