# Supplementary material for: The α2AR/Caveolin‐1/p38MAPK/NF‐κB axis explains dexmedetomidine protection against lung injury following intestinal ischaemia‐reperfusion
Source: J Cell Mol Med. 2021 Jun 10;25(13):6361–72. doi: 10.1111/jcmm.16614 (PMC8406475; doi:10.1111/jcmm.16614)
Supplement: Supplementary file 1 — Figure S1 [file JCMM-25-6361-s001.docx]

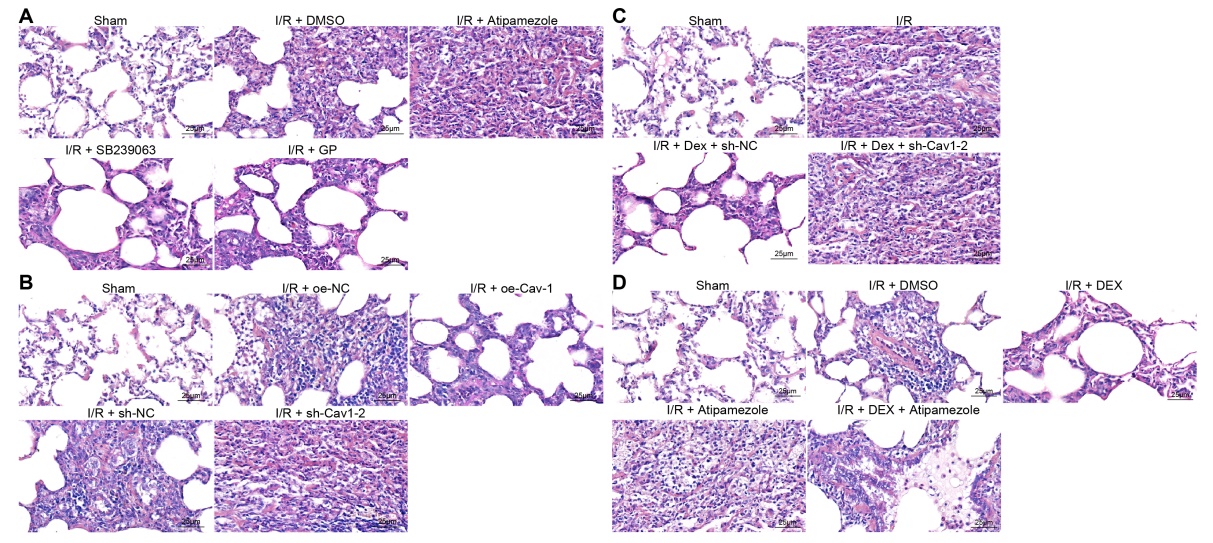


**Supplementary Figure 1** Representative images of H&E staining. A, Histopathological examination of rat lung tissues with injections of Atipamezole, SB239063, and GP inhibitors prior to intestinal I/R injury (×400, scale bar = 25 μm); B, Histopathological examination of rat lung tissues with Cav-1 overexpression or knockdown before intestinal I/R injury (×400, scale bar = 25 μm); C, Histopathological examination of rat lung tissues upon Dex treatment and/or Cav-1 knockdown prior to intestinal I/R injury by H&E staining (×400, scale bar = 25 μm); D, Histopathological examination of rat lung tissues after combination treatment of Dex and Atipamezole prior to intestinal I/R injury by H&E staining (×400, scale bar = 25 μm).
