# Supplementary material for: The α2AR/Caveolin‐1/p38MAPK/NF‐κB axis explains dexmedetomidine protection against lung injury following intestinal ischaemia‐reperfusion
Source: J Cell Mol Med. 2021 Jun 10;25(13):6361–72. doi: 10.1111/jcmm.16614 (PMC8406475; doi:10.1111/jcmm.16614)
Supplement: Supplementary file 2 — Figure S2 [file JCMM-25-6361-s004.docx]

**
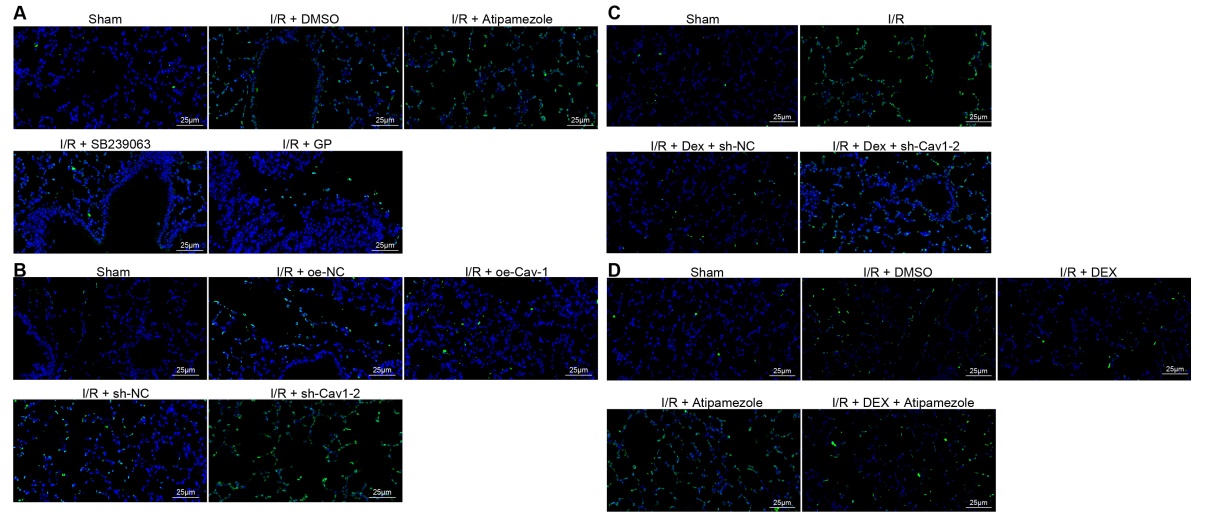
**

**Supplementary Figure 2** Representative images of TUNEL staining. A, Apoptosis of rat lung tissues with injections of Atipamezole, SB239063, and GP inhibitors prior to intestinal I/R injury, detected by TUNEL staining (×400, scale bar = 25 μm); B, Apoptosis of rat lung tissues with Cav-1 overexpression or knockdown before intestinal I/R injury, detected by TUNEL staining (×400, scale bar = 25 μm); C, Apoptosis of rat lung tissues upon Dex treatment and/or Cav-1 knockdown prior to intestinal I/R injury, detected by TUNEL staining (×400, scale bar = 25 μm); D, Apoptosis of rat lung tissues after combination treatment of Dex and Atipamezole prior to intestinal I/R injury, detected by TUNEL staining (×400, scale bar = 20 μm).
