# Supplementary material for: The α2AR/Caveolin‐1/p38MAPK/NF‐κB axis explains dexmedetomidine protection against lung injury following intestinal ischaemia‐reperfusion
Source: J Cell Mol Med. 2021 Jun 10;25(13):6361–72. doi: 10.1111/jcmm.16614 (PMC8406475; doi:10.1111/jcmm.16614)
Supplement: Supplementary file 3 — Figure S3 [file JCMM-25-6361-s003.docx]

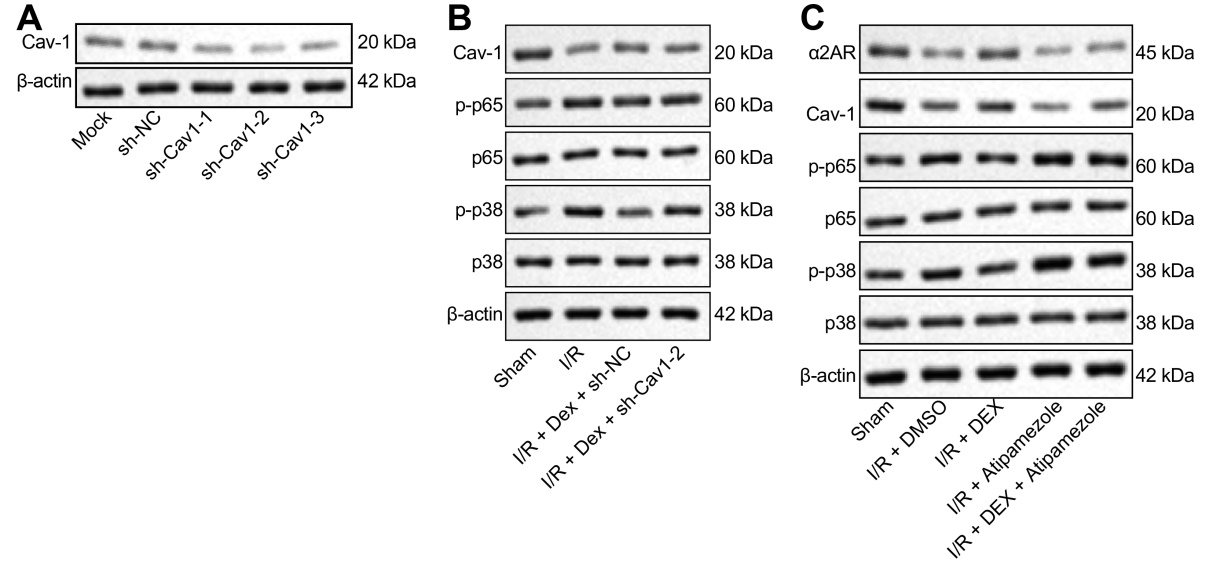


**Supplementary Figure 3** Protein bands of Western blot analysis. A, Protein bands for Figure 4A; B, Protein bands for Figure 4H; C, Protein bands for Figure 5A
