# Supplementary material for: The α2AR/Caveolin‐1/p38MAPK/NF‐κB axis explains dexmedetomidine protection against lung injury following intestinal ischaemia‐reperfusion
Source: J Cell Mol Med. 2021 Jun 10;25(13):6361–72. doi: 10.1111/jcmm.16614 (PMC8406475; doi:10.1111/jcmm.16614)
Supplement: Supplementary file 4 — Table S1‐S2 [file JCMM-25-6361-s002.docx]

**Supplementary Table 1** Mortality rate of rats in each group

| Groups | Death (No.) | Mortality rate (%) |
| --- | --- | --- |
| Sham | 0/12 | 0 |
| I/R | 5/12 | 41.67 |
| I/R + DMSO | 5/12 | 41.67 |
| I/R + DEX | 2/12 | 16.67 |
| I/R + Atipamezole | 7/12 | 58.33 |
| I/R + SB239063 | 3/12 | 25 |
| I/R + Gypenoside | 2/12 | 16.67 |
| I/R + DEX + Atipamezole | 4/12 | 33.33 |
| I/R + sh-NC | 5/12 | 41.67 |
| I/R + sh-Cav-1 | 8/12 | 66.67 |
| I/R + oe-NC | 5/12 | 41.67 |
| I/R + oe-Cav-1 | 1/12 | 8.33 |
| I/R + DEX + sh-Cav-1 | 6/12 | 50 |
| I/R + DEX + sh-NC | 3/12 | 25 |

**Supplementary Table 2** Primer sequences for reverse transcription quantitative polymerase chain reaction

| Gene | Primer sequence |
| --- | --- |
| α_2A_-AR | Forward 5’-ACACTCGAGGGATCCTGGCCTCTCTCGGATC-3’ |
|  | Reverse 5’-ACAAAGCTTGGGCGCAAAGCTGCCCTCGG-3’ |
| Caveolin 1 | Forward 5’-CGTAGGTTCCTAGCCCCCTGA-3’ |
|  | Reverse 5’-GTACAAGTGCAATGGGTCAAA-3’ |
| GAPDH | Forward 5’-ATGCCATCACTGCCACTCA-3’ |
|  | Reverse 5’-CCTGCTTCACCACCTTCTTG-3’ |
